# New Appliances and Things Medical

**Published:** 1903-05-09

**Authors:** 


### NEW APPLIANCES AND THINGS MEDICAL.

**[We shall be glad to receive at our Office, 28 & 29 Southampton Street, Strand, London, W.C., from the manufacturers, specimens of all new preparations and appliances which may be brought out from time to time.]**

### "THE HOME MASSEUR."

(Heath and George, 16 Goswell Road, London, E.C.)

This is a simple and practical apparatus for carrying out home massage. It is a modification of the ordinary shot-bag or cannon-ball weight masseur. In the treatment of constipation and neurasthenic disorders, a treatment which, by-the-bye, must be long-continued to be of any practical benefit, a simple and inexpensive means of applying massage is of the greatest importance. Combined with deep-breathing exercises, such as are recommended in a little brochure supplied by the manufacturers, we believe that massage systematically applied by this ingenious home masseur will be a great boon to a large number of sufferers from chronic constipation, want of muscular tone, anæmia, phthisis, and general neurasthenia. The price of the home masseur is, we understand, 12s. 6d.

### POCKET SPITTOON.

(Allen and Hanburys, Ltd., Bethnal Green, E.)

This new pocket spittoon is a cleanly and hygienic receptacle for the sputum of phthisical patients. It is made of aluminium, is very light, and can easily be disinfected by antiseptics. The lid fits accurately with a snap, and the spittoon can be conveniently carried in the pocket.

